# Beta-thalassemia

**DOI:** 10.1186/1750-1172-5-11

**Published:** 2010-05-21

**Authors:** Renzo Galanello, Raffaella Origa

**Affiliations:** 1 Dipartimento di Scienze Biomediche e Biotecnologie- Università di Cagliari, Ospedale Regionale, Microcitemie ASL Cagliari, Cagliari, Italy

## Abstract

Beta-thalassemias are a group of hereditary blood disorders characterized by anomalies in the synthesis of the beta chains of hemoglobin resulting in variable phenotypes ranging from severe anemia to clinically asymptomatic individuals. The total annual incidence of symptomatic individuals is estimated at 1 in 100,000 throughout the world and 1 in 10,000 people in the European Union. Three main forms have been described: thalassemia major, thalassemia intermedia and thalassemia minor. Individuals with thalassemia major usually present within the first two years of life with severe anemia, requiring regular red blood cell (RBC) transfusions. Findings in untreated or poorly transfused individuals with thalassemia major, as seen in some developing countries, are growth retardation, pallor, jaundice, poor musculature, hepatosplenomegaly, leg ulcers, development of masses from extramedullary hematopoiesis, and skeletal changes that result from expansion of the bone marrow. Regular transfusion therapy leads to iron overload-related complications including endocrine complication (growth retardation, failure of sexual maturation, diabetes mellitus, and insufficiency of the parathyroid, thyroid, pituitary, and less commonly, adrenal glands), dilated myocardiopathy, liver fibrosis and cirrhosis). Patients with thalassemia intermedia present later in life with moderate anemia and do not require regular transfusions. Main clinical features in these patients are hypertrophy of erythroid marrow with medullary and extramedullary hematopoiesis and its complications (osteoporosis, masses of erythropoietic tissue that primarily affect the spleen, liver, lymph nodes, chest and spine, and bone deformities and typical facial changes), gallstones, painful leg ulcers and increased predisposition to thrombosis. Thalassemia minor is clinically asymptomatic but some subjects may have moderate anemia. Beta-thalassemias are caused by point mutations or, more rarely, deletions in the beta globin gene on chromosome 11, leading to reduced (beta^+^) or absent (beta^0^) synthesis of the beta chains of hemoglobin (Hb). Transmission is autosomal recessive; however, dominant mutations have also been reported. Diagnosis of thalassemia is based on hematologic and molecular genetic testing. Differential diagnosis is usually straightforward but may include genetic sideroblastic anemias, congenital dyserythropoietic anemias, and other conditions with high levels of HbF (such as juvenile myelomonocytic leukemia and aplastic anemia). Genetic counseling is recommended and prenatal diagnosis may be offered. Treatment of thalassemia major includes regular RBC transfusions, iron chelation and management of secondary complications of iron overload. In some circumstances, spleen removal may be required. Bone marrow transplantation remains the only definitive cure currently available. Individuals with thalassemia intermedia may require splenectomy, folic acid supplementation, treatment of extramedullary erythropoietic masses and leg ulcers, prevention and therapy of thromboembolic events. Prognosis for individuals with beta-thalassemia has improved substantially in the last 20 years following recent medical advances in transfusion, iron chelation and bone marrow transplantation therapy. However, cardiac disease remains the main cause of death in patients with iron overload.

## Disease name and synonyms

The term thalassemia is derived from the Greek, thalassa (sea) and haima (blood). Beta-thalassemia includes three main forms: Thalassemia Major, variably referred to as "Cooley's Anemia" and "Mediterranean Anemia", Thalassemia Intermedia and Thalassemia Minor also called "beta-thalassemia carrier", "beta-thalassemia trait" or "heterozygous beta-thalassemia". Apart from the rare dominant forms, subjects with thalassemia major are homozygotes or compound heterozygotes for beta^0 ^or beta^+ ^genes, subjects with thalassemia intermedia are mostly homozygotes or compound heterozygotes and subjects with thalassemia minor are mostly heterozygotes.

### Definition

Beta-thalassemia syndromes are a group of hereditary blood disorders characterized by reduced or absent beta globin chain synthesis, resulting in reduced Hb in red blood cells (RBC), decreased RBC production and anemia. Most thalassemias are inherited as recessive traits. Beta-thalassemias can be classified into:

- Beta-thalassemia

• Thalassemia major

•Thalassemia intermedia

•Thalassemia minor

- Beta-thalassemia with associated Hb anomalies

• HbC/Beta-thalassemia

• HbE/Beta-thalassemia

• HbS/Beta-thalassemia (clinical condition more similar to sickle cell disease than to thalassemia major or intermedia)

- Hereditary persistence of fetal Hb and beta-thalassemia

- Autosomal dominant forms

- Beta-thalassemia associated with other manifestations

• Beta-thalassemia-tricothiodystrophy

• X-linked thrombocytopenia with thalassemia

### Epidemiology

Beta-thalassemia is prevalent in Mediterranean countries, the Middle East, Central Asia, India, Southern China, and the Far East as well as countries along the north coast of Africa and in South America. The highest carrier frequency is reported in Cyprus (14%), Sardinia (10.3%), and Southeast Asia [1]. The high gene frequency of beta-thalassemia in these regions is most likely related to the selective pressure from *Plasmodium falciparum *malaria [1]. Population migration and intermarriage between different ethnic groups has introduced thalassemia in almost every country of the world, including Northern Europe where thalassemia was previously absent. It has been estimated that about 1.5% of the global population (80 to 90 million people) are carriers of beta-thalassemia, with about 60,000 symptomatic individuals born annually, the great majority in the developing world. The total annual incidence of symptomatic individuals is estimated at 1 in 100,000 throughout the world and 1 in 10,000 people in the European Union. However, accurate data on carrier rates in many populations are lacking, particularly in areas of the world known or expected to be heavily affected [2]. According to Thalassemia International Federation, only about 200,000 patients with thalassemia major are alive and registered as receiving regular treatment around the world [3]. The most common combination of beta-thalassemia with abnormal Hb or structural Hb variant with thalassemic properties is HbE/beta-thalassemia which is most prevalent in Southeast Asia where the carrier frequency is around 50%.

## Clinical description

The phenotypes of homozygous or genetic heterozygous compound beta-thalassemias include thalassemia major and thalassemia intermedia. Individuals with thalassemia major usually come to medical attention within the first two years of life and require regular RBC transfusions to survive. Thalassemia intermedia includes patients who present later and do not require regular transfusion. Except in the rare dominant forms, heterozygous beta-thalassemia results in the clinically silent carrier state. HbE/beta-thalassemia and HbC/beta-thalassemia exhibit a great range in terms of diversity of phenotypes and spectrum of severity.

### Beta-thalassemia major

Clinical presentation of thalassemia major occurs between 6 and 24 months. Affected infants fail to thrive and become progressively pale. Feeding problems, diarrhea, irritability, recurrent bouts of fever, and progressive enlargement of the abdomen caused by spleen and liver enlargement may occur. In some developing countries, where due to the lack of resources patients are untreated or poorly transfused, the clinical picture of thalassemia major is characterized by growth retardation, pallor, jaundice, poor musculature, genu valgum, hepatosplenomegaly, leg ulcers, development of masses from extramedullary hematopoiesis, and skeletal changes resulting from expansion of the bone marrow. Skeletal changes include deformities in the long bones of the legs and typical craniofacial changes (bossing of the skull, prominent malar eminence, depression of the bridge of the nose, tendency to a mongoloid slant of the eye, and hypertrophy of the maxillae, which tends to expose the upper teeth).

If a regular transfusion program that maintains a minimum Hb concentration of 9.5 to 10.5 g/dL is initiated, growth and development tends to be normal up to 10 to 12 years [3]. Transfused patients may develop complications related to iron overload. Complications of iron overload in children include growth retardation and failure or delay of sexual maturation. Later iron overload-related complications include involvement of the heart (dilated myocardiopathy or rarely arrythmias), liver (fibrosis and cirrhosis), and endocrine glands (diabetes mellitus, hypogonadism and insufficiency of the parathyroid, thyroid, pituitary, and, less commonly, adrenal glands) [4]. Other complications are hypersplenism, chronic hepatitis (resulting from infection with viruses that cause hepatitis B and/or C), HIV infection, venous thrombosis, and osteoporosis. The risk for hepatocellular carcinoma is increased in patients with liver viral infection and iron overload [5]. Compliance with iron chelation therapy (see later) mainly influences frequency and severity of the iron overload-related complications. Individuals who have not been regularly transfused usually die before the second-third decade. Survival of individuals who have been regularly transfused and treated with appropriate chelation extends beyond age of 40 years. Cardiac disease caused by myocardial siderosis is the most important life-limiting complication of iron overload in beta-thalassemia. In fact, cardiac complications are the cause of the deaths in 71% of the patients with beta-thalassemia major [6].

### Beta-thalassemia intermedia

Individuals with thalassemia intermedia present later than thalassemia major, have milder anemia and by definition do not require or only occasionally require transfusion. At the severe end of the clinical spectrum, patients present between the ages of 2 and 6 years and although they are capable of surviving without regular blood transfusion, growth and development are retarded. At the other end of the spectrum are patients who are completely asymptomatic until adult life with only mild anemia. Hypertrophy of erythroid marrow with the possibility of extramedullary erythropoiesis, a compensatory mechanism of bone marrow to overcome chronic anemia, is common. Its consequences are characteristic deformities of the bone and face, osteoporosis with pathologic fractures of long bones and formation of erythropoietic masses that primarily affect the spleen, liver, lymph nodes, chest and spine. Enlargement of the spleen is also a consequence of its major role in clearing damaged red cells from the bloodstream. Extramedullary erythropoiesis may cause neurological problems such as spinal cord compression with paraplegia and intrathoracic masses. As a result of ineffective erythropoiesis and peripheral hemolysis, thalassemia intermedia patients may develop gallstones, which occur more commonly than in thalassemia major [7]. Patients with thalassemia intermedia frequently develop leg ulcers and have an increased predisposition to thrombosis as compared to thalassemia major, especially if splenectomised. Such events include deep vein thrombosis, portal vein thrombosis, stroke and pulmonary embolism [8].

Although individuals with thalassemia intermedia are at risk of iron overload secondary to increased intestinal iron absorption, hypogonadism, hypothyroidism and diabetes are not common [9]. Women may have successful spontaneous pregnancies. However, if blood transfusions are necessary during pregnancy, those never or minimally transfused are at risk of developing hemolytic alloantibodies and erythrocyte autoantibodies. Intrauterine growth retardation, despite a regular transfusion regimen, has been reported [10]. Cardiac involvement in thalassemia intermedia results mainly from a high-output state and pulmonary hypertension, while systolic left ventricle function is usually preserved [11]. Pseudoxantoma elasticum, a diffuse connective tissue disorder with vascular manifestation caused by degeration of the elastic lamina of the arterial wall and calcium deposition, has been described in such patients [12].

### Beta-thalassemia minor

Carriers of thalassemia minor are usually clinically asymptomatic but sometimes have a mild anemia. When both parents are carriers there is a 25% risk at each pregnancy of having children with homozygous thalassemia.

### Dominant beta-thalassemia

In contrast with the classical recessive forms of beta-thalassemia, which lead to a reduced production of normal beta globin chains, some rare mutations result in the synthesis of extremely unstable beta globin variants which precipitate in erythroid precursors causing ineffective erythropoiesis. These mutations are associated with a clinically detectable thalassemia phenotype in the heterozygote and are therefore referred to as dominant beta-thalassemias [13]. The presence of hyper-unstable Hb should be suspected in any individual with thalassemia intermedia when both parents are hematologically normal, or in families with a pattern of autosomal dominant transmission of the thalassemia intermedia phenotype. Beta globin gene sequencing establishes the diagnosis.

### Beta-thalassemia associated with other Hb anomalies

The interaction of HbE and beta-thalassemia results in thalassemia phenotypes ranging from a condition indistinguishable from thalassemia major to a mild form of thalassemia intermedia. Depending on the severity of symptoms three categories may be identified:

- Mild HbE/beta-thalassemia: It is observed in about 15% of all cases in Southeast Asia. This group of patients maintains Hb levels between 9 and 12 g/dl and usually does not develop clinically significant problems. No treatment is required.

- Moderately severe HbE/beta-thalassemia: The majority of HbE/beta-thalassemia cases fall into this category. The Hb levels remain at 6-7 g/dl and the clinical symptoms are similar to thalassemia intermedia. Transfusions are not required unless infections precipitate further anemia. Iron overload may occur.

- Severe HbE/beta-thalassemia: The Hb level can be as low as 4-5 g/dl. Patients in this group manifest symptoms similar to thalassemia major and are treated as thalassemia major patients.

Patients with HbC/beta-thalassemia may live free of symptoms and be diagnosed during routine tests. When present, clinical manifestations are anemia and enlargement of the spleen. Blood transfusions are seldom required. Microcytosis and hypochromia are found in every case. The blood film shows distinctive Hb C crystals with straight parallel edges, target cells, and irregularly contracted cells with features of thalassemia such as microcytosis.

The association of hereditary persistence of fetal Hb (HPFH) with beta-thalassemia mitigates the clinical manifestations which vary from normal to thalassemia intermedia.

Individuals with HbS/beta-thalassemia have a clinical course similar to that of Hb SS.

### Beta-thalassemia associated with other features

In rare instances the beta-thalassemia defect does not lie in the beta globin gene cluster. In cases in which the beta-thalassemia trait is associated with other features, the molecular lesion has been found either in the gene encoding the transcription factor TFIIH (beta-thalassemia trait associated with tricothiodystrophy) or in the X-linked transcription factor GATA-1 (X-linked thrombocytopenia with thalassemia) [14,15].

## Etiology

More than 200 mutations have been so far reported; the large majority are point mutations in functionally important regions of the beta globin gene [16,17]. Deletions of the beta globin gene are uncommon. The beta globin gene mutations cause a reduced or absent production of beta globin chains. A list of common mutations according to the severity and ethnic distribution is reported in Table 1.

**Table 1:** Common types of beta-thalassemia: severity and ethnic distribution.

Population	β-gene mutation	Severity
**Indian**	-619 del	β^0^

**Mediterranean**	-101 C→T	β^++^

**Black**	-88 C→T	β^++^

**Mediterranean; African**	-87 C→G	β^++^

**Japanese**	-31 A→G	β^++^

**African**	-29 A→G	β^++^

**Southeast Asian**	-28 A→C	β^++^

**Mediterranean; Asian Indian**	IVS1-nt1 G→A	β^0^

**East Asian; Asian Indian**	IVS1-nt5 G→C	β^0^

**Mediterranean**	IVS1-nt6 T→C	β^+/++^

**Mediterranean**	IVS1-nt110 G→A	β^+^

**Chinese**	IVS2-nt654 C→T	β^+^

**Mediterranean**	IVS2-nt745 C→G	β^+^

**Mediterranean**	codon 39 C→T	β^0^

**Mediterranean**	codon 5 -CT	β^0^

**Mediterranean; African-American**	codon 6 -A	β^0^

**Southeast Asian**	codon 41/42 -TTCT	β^0^

**African-American**	AATAAA to AACAAA	β^++^

**Mediterranean**	AATAAA to AATGAA	β^++^

**Mediterranean**	codon 27 G→T Hb (Hb Knossos)	β^++^

**Southeast Asian**	codon 79 G>A (Hb E)	β^++^

**Malaysia**	Codon 19 G>A (Hb Malay)	

β^0^:complete absence of beta globin on the affected allele

β^+^:residual production of beta globin (around 10%)

β^++^:very mild reduction in beta globin production

### Genetic modifiers

Modifier genes are defined as genetic variants that lead to differences in disease phenotype. In homozygous beta-thalassemia, primary genetic modifiers, affecting the clinical severity of the disease, include genetic variants able to reduce the globin chain imbalance, therefore resulting in a milder form of thalassemia. These factors are the presence of silent or mild beta-thalassemia alleles associated with a high residual output of beta globin, the co-inheritance of alpha thalassemia and/or of genetic determinants able to sustain a continuous production of gamma globin chains (HbF) in adult life [18]. Some beta-thalassemia mutations (i.e. deletion and non deletion delta beta-thalassemia, deletions of the 5' region of the beta globin gene) increase *"per se" *the gamma globin gene output. Other mutations increasing HbF production are those associated with deletional and non-deletional HPFH linked to the beta globin gene cluster. Recently, the genome-wide association approach, particularly studying quantitative trait loci (QTL) which cause elevated HbF, have revealed genetic elements (i.e. polymorphism in BCL11A gene and in the HBS1LCMYB intergenic region) unlinked to beta globin gene cluster, able to modify the severity of the homozygous beta zero thalassemia [19].

The clinical phenotype of homozygous beta-thalassemia may also be modified by the co-inheritance of other genetic variants mapping outside the globin clusters. These secondary genetic modifiers influence mainly the complications of the thalassemia phenotype. Several secondary genetic modifiers have been identified in the recent years. The presence of (TA)_7 _polymorphism in the promoter region of the uridine diphosphate-glucuronosyltransferase gene, which in the homozygous state is associated with the Gilbert syndrome, is a risk factor for the development of cholelitiasis in thalassemia major and intermedia patients [20,21]. Other candidate genes for modification of the thalassemia phenotype are the apolipoprotein E ε4 allele and some HLA haplotypes, which seem to be genetic risk factors for left ventricular failure in homozygous beta-thalassemia [22,23]. Less consistent data have been reported for genes involved in iron metabolism (i.e. C282Y and H63D HFE gene mutations), probably because their effect on iron overload is hidden as a result of treatment (i.e. secondary iron overload from red cell transfusion and iron chelation), and for genes associated with bone metabolism [24-26]. Recently, a polymorphism in glutathione-Stransferase M1 gene has been associated with an increased risk of heart iron overload in thalassemia major [27].

In some instances, heterozygous beta-thalassemia may lead to the thalassemia intermedia phenotype instead of the asymptomatic carrier state. Most of these patients have excess functional alpha globin genes (alpha gene triplication or quadruplication) which increases the imbalance in the ratio of alpha/non-alpha globin chain synthesis [18,28].

### Pathophysiology

The reduced amount (beta^+^) or absence (beta^0^) of beta globin chains result in a relative excess of unbound alpha globin chains that precipitate in erythroid precursors in the bone marrow, leading to their premature death and hence to ineffective erythropoiesis. The degree of globin chain reduction is determined by the nature of the mutation at the beta globin gene located on chromosome 11.

Peripheral hemolysis contributing to anemia is less prominent in thalassemia major than in thalassemia intermedia, and occurs when insoluble alpha globin chains induce membrane damage to the peripheral erythrocytes. Anemia stimulates the production of erythropoietin with consequent intensive but ineffective expansion of the bone marrow (up 25 to 30 times normal), which in turn causes the typical previously described bone deformities. Prolonged and severe anemia and increased erythropoietic drive also result in hepatosplenomegaly and extramedullary erythropoiesis.

### Hereditary transmission

The beta-thalassemias are inherited in an autosomal recessive manner. The parents of an affected child are obligate heterozygotes and carry a single copy of a disease-causing beta globin gene mutation. At conception, each child of heterozygotes parents has 25% chance of being affected, 50% chance of being an asymptomatic carrier, and 25% chance of being unaffected and not carrier. The parents of the proband have a 1 in 4 (25%) risk of having further affected children in each pregnancy.

Dominant forms of beta-thalassemia, associated with mutations that result in the production of highly unstable beta globulin variants and leading to a clinically manifesting phenotype of beta-thalassemia in heterozygotes, have been discussed above in the clinical description section [13].

## Diagnosis

### Clinical Diagnosis

Thalassemia major is usually suspected in an infant younger than two years of age with severe microcytic anemia, mild jaundice and hepatosplenomegaly. Thalassemia intermedia presents at a later age with similar but milder clinical findings. Carriers are usually asymptomatic, but sometimes may have mild anemia.

### Hematologic Diagnosis

**RBC indices **show microcytic anemia. Thalassemia major is characterized by reduced Hb level (<7 g/dl), mean corpuscolar volume (MCV) > 50 < 70 fl and mean corpuscolar Hb (MCH) > 12< 20 pg. Thalassemia intermedia is characterized by Hb level between 7 and 10 g/dl, MCV between 50 and 80 fl and MCH between 16 and 24 pg. Thalassemia minor is characterized by reduced MCV and MCH, with increased Hb A^2 ^level [29].

## Peripheral blood smear

• Affected individuals show RBC morphologic changes [microcytosis, hypochromia, anisocytosis, poikilocytosis (spiculated tear-drop and elongated cells)], and nucleated RBC (i.e., erythroblasts). The number of erythroblasts is related to the degree of anemia and is markedly increased after splenectomy.

• Carriers have less severe RBC morphologic changes than affected individuals. Erythroblasts are normally not seen.

**Qualitative and quantitative Hb analysis **(by cellulose acetate electrophoresis and DE-52 microchromatography or HPLC) identifies the amount and type of Hb present.

The Hb pattern in beta-thalassemia varies according to beta-thalassemia type. In beta^0 ^thalassemia, homozygotes HbA is absent and HbF constitutes the 92-95% of the total Hb. In beta^+ ^thalassemia homozygotes and beta^+^/beta^0 ^genetic compounds HbA levels are between 10 and 30% and HbF between 70-90%. HbA2 is variable in beta thalassemia homozygotes and it is enhanced in beta thalassemia minor.

Hb electrophoresis and HPLC also detect other hemoglobinopathies (S, C, E, O_Arab_, Lepore) that may interact with beta-thalassemia.

### Molecular Genetic Analysis

• The prevalence of a limited number of mutations in each population has greatly facilitated molecular genetic testing.

Commonly occurring mutations of the beta globin gene are detected by PCR-based procedures [30]. The most commonly used methods are reverse dot blot analysis or primer-specific amplification, with a set of probes or primers complementary to the most common mutations in the population from which the affected individual originated.

• If targeted mutation analysis fails to detect the mutation, beta globin gene sequence analysis can be used to detect mutations in the beta globin gene.

## Differential diagnosis

Few conditions share similarities with homozygous beta-thalassemia:

• The genetically-determined sideroblastic anemias are easily differentiated because of ring sideroblasts in the bone marrow and variably elevated serum concentration of erythrocyte protoporphyrin. Most sideroblastic anemias are associated with defects in the heme biosynthetic pathway, especially delta-aminolevulinic acid synthase.

• Congenital dyserythropoietic anemias do not have high HbF and do have other distinctive features, such as multinuclearity of the red blood cell precursors.

• A few acquired conditions associated with high HbF (juvenile chronic myelomonocytic leukemia with normal kariotype, aplastic anemia both congenital and acquired during the recovery phase) may be mistaken for beta-thalassemia, even though they have very characteristic clinical and hematological features.

Typical beta-thalassemia carriers are identified by analysis of RBC indices, which shows microcytosis (low MCV) and reduced content of Hb per red cell (low MCH), and by qualitative and quantitative Hb analysis, which displays the increase of HbA2.

Pitfalls in carrier identification by hematologic testing are:

• *Coinheritance of alpha-thalassemia*, which may normalize the RBC indices. However, in alpha/beta double heterozygotes, the HbA2 concentration remains in the beta-thalassemia carrier range and thus is diagnostic. Therefore, HbA^2 ^determination should always be performed for betathalassemia carrier identification.

• *Coinheritance of delta-thalassemia*, which may reduce to normal the increased Hb A2 levels typical of the beta-thalassemia carrier state. Double heterozygosity for delta- and betathalassemia can be distinguished from the most common alpha-thalassemia carrier state by globin chain synthesis or globin gene analysis.

• *Silent mutations*, i.e., very mild mutations associated with consistent residual output of Hb beta chains and with normal RBC indices and normal or borderline HbA2. The above reported groups of carriers are referred to as atypical carriers (see Table 1 β^++ ^mutations).

When the hematologic analysis is abnormal, molecular genetic testing of beta globin gene is performed to identify the disease-causing mutation [30].

**Genetic counseling and prenatal diagnosis **Prevention of beta-thalassemia is based on carrier identification, genetic counseling and prenatal diagnosis [31]. Carrier detection has been previously described. Genetic counseling provides information for individuals and at risk couples (i.e. both carriers) regarding the mode of inheritance, the genetic risk of having affected children and the natural history of the disease including the available treatment and therapies under investigation. Prenatal diagnosis for pregnancies at increased risk is possible by analysis of DNA extracted from fetal cells obtained by amniocentesis, usually performed at approximately 15-18 weeks' gestation or chorionic villi sampling at 11 weeks' gestation. Both disease-causing alleles must be identified before prenatal testing can be performed. Analysis of fetal cells in maternal blood and analysis of fetal DNA in maternal plasma for the presence of the father's mutation are currently under investigation [32,33]. Preimplantation genetic diagnosis may be available for families in which the disease-causing mutations have been identified.

## Management of thalassemia major

### Transfusions

The goals of transfusion therapy are correction of anemia, suppression of erythropoiesis and inhibition of gastrointestinal iron absorption, which occurs in non transfused patients as a consequence of increased, although ineffective, erythropoiesis. The decision to start transfusion in patients with confirmed diagnosis of thalassemia should be based on the presence of severe anemia (Hb < 7 g/dl for more than two weeks, excluding other contributory causes such as infections). However, also in patients with Hb > 7 g/dl, other factors should be considered, including facial changes, poor growth, evidence of bony expansion and increasing splenomegaly. When possible, the decision to start regular transfusions should not be delayed until after the second- third year, due to the risk of developing multiple red cell antibodies and subsequent difficulty in finding suitable blood donors. Several different transfusional regimens have been proposed over the years, but the most widely accepted aims at a pre-transfusional Hb level of 9 to 10 g/dl and a post-transfusion level of 13 to 14 g/dl. This prevents growth impairment, organ damage and bone deformities, allowing normal activity and quality of life [3,4]. The frequency of transfusion is usually every two to four weeks. Shorter intervals might further reduce the overall blood requirement, but are incompatible with an acceptable quality of life. The amount of blood to be transfused depends on several factors including weight of the patient, target increase in Hb level and hematocrit of blood unit. Appropriate graphs and formulae to calculate the amount of blood to be transfused are available [3]. In general, the amount of transfused RBC should not exceed 15 to 20 ml/kg/day, infused at a maximum rate of 5 ml/kg/hour, to avoid a fast increase in blood volume. To monitor the effectiveness of transfusion therapy, some indices should be recorded at each transfusion, such as pre- and post-transfusion Hb, amount and hematocrit of the blood unit, daily Hb fall and transfusional interval. These measurements enable two important parameters to be calculated: red cell requirement and iron intake. Dedicated computerized programs (Webthal) are available to monitor transfused thalassemia patients accurately [34]. Although red cell transfusions are lifesavers for patients with thalassemia, they are responsible for a series of complications and expose the patients to a variety of risks. Iron overload is the most relevant complication associated with transfusion therapy. Other adverse events associated with red cell transfusions are summarized in Table 2.

**Table 2:** Transfusion-dependent complications.

Iron overload	
Infections	
	*Known*
	*- Viral (HIV, HCV, HBV, HTLVI, West Nile virus)*
	*- Bacterial*
	*- Parasitic*
	*Rare*
	*- Creutzfeld-Jacob disease*
	*- Emerging and new pathogens*
Hemolytic reactions	
	*Acute hemolytic reactions*
	*Delayed hemolytic reactions*
	*Autoimmune hemolytic anemia*

Non-Hemolytic reactions	
	*Allergic and anaphylactic reactions*
	*Febrile non-hemolytic reactions*
	*Transfusion-related acute lung injury (TRALI)*
	*Transfusion-associated graft-versus-host disease*
	*Circulatory overload*
	*Post-transfusion purpura*

### Assessment and treatment of Iron overload

Patients maintained on a regular transfusion regimen progressively develop clinical manifestations of iron overload: hypogonadism (35-55% of the patients), hypothyroidism (9-11%), hypoparathyroidism (4%), diabetes (6-10%), liver fibrosis, and heart dysfunction (33%) [35,36]. Iron status should be accurately assessed in order to evaluate its clinical relevance, the need for treatment, and the timing and monitoring of chelation therapy. The iron status of multitransfused patients can be assessed by several methods. Serum ferritin has in general been found to correlate with body iron stores [37]. However, as a single value it is not always reliable because, being an acute-phase reactant, it is influenced by other factors such as inflammatory disorders, liver disease, malignancy. Despite this, serial measurements of serum ferritin remain a reliable and the easiest method to evaluate iron overload and efficacy of chelation therapy. Determination of liver iron concentration in a liver biopsy specimen shows a high correlation with total body iron accumulation and is considered the gold standard for the evaluation of iron overload [38]. However, liver biopsy is an invasive technique with the possibility (though low) of complications. Moreover, we should consider that the presence of hepatic fibrosis, which commonly occurs in individuals with iron overload and HCV infection, and heterogeneous liver iron distribution can lead to possible false negative results [39]. In recent years, nuclear magnetic resonance imaging (MRI) techniques for assessing iron loading in the liver and heart have been introduced [40-43]. R2 and T2* parameters have been validated for liver iron concentration. Cardiac T2* is reproducible, transferable between different scanners, correlates with cardiac function, and relates to tissue iron concentration. Clinical utility of T2* in monitoring patients with siderotic cardiomyopathy has been demonstrated [44,45]. Calibration of T2* in the heart will be available in the near future. Magnetic biosusceptometry (SQUID), is another option for a reliable measurement of hepatic iron concentration [46]; however, magnetic susceptometry is presently available only in a limited number of centers worldwide.

As the body has no effective means for removing iron, the only way to remove excess iron is to use iron binders (chelators), which allow iron excretion through the urine and/or stool. As a general rule, patients should start iron chelation treatment once they have had 10-20 transfusions or when ferritin levels rise above 1000 ng/ml [3]. The first drug available for treatment of iron overload was *deferoxamine *(DFO), an exadentate iron chelator that is not orally absorbed and thus needs parenteral administration, usually as a subcutaneous 8- to 12-hour nightly infusion, 5-7 nights a week. Average dosage is 20-40 mg/kg body weight for children and 30-50 mg/kg body weight for adults [3,4]. In high risk cases, continuous administration of DFO via an implanted delivery system (Port-a-cath) or subcutaneously, at doses between 50 and 60 mg/kg per day, were the only options to intensify the chelation treatment before the advent of the combined therapy with DFO and deferiprone [44]. Implanted delivery systems are associated with risk of thrombosis and infection. With DFO, iron is excreted both in faeces (about 40%) and in urine. The most frequent adverse effects of DFO are local reactions at the site of infusion, such as pain, swelling, induration, erythema, burning, pruritus, wheals and rash, occasionally accompanied by fever, chills and malaise. Other complications, mainly associated with high doses of DFO in young patients and low ferritin values are:

• sensorineural hypoacusia, particularly at high frequencies

• ocular toxicity (night-blindness, blurred vision, decreased visual acuity, impairment of colour vision, cataract and other disturbances of the eye)

• retarded growth and skeletal changes with a disproportionately short trunk and dysplasia of the long bones

• infections by *Yersinia Enterocolitica*, and other pathogens (*Klebsiella Pneumoniae*).

It is therefore important to monitor patients receiving DFO regularly with audiometric and ophthalmologic tests and with regular evaluation of growth and bone changes.

The use of DFO decreases morbidity and mortality among those who are able to comply with regular prolonged infusions [47]. However, because of the side effects and the inconvenient parenteral administration, a consistent proportion of patients is non-compliant, limiting the usefulness of this chelator [35].

The orphan drug *deferiprone *(DFP) is an orally active iron chelator which has emerged from an extensive search for new treatment of iron overload. Comparative studies have shown that this chelator, at doses of 75-100 mg/kg/day may be as effective as DFO in removing body iron [48]. Retrospective and prospective studies have shown that DFP monotherapy is significantly more effective than deferoxamine in decreasing myocardial siderosis in thalassemia major [49-51]. Agranulocytosis is the most serious side effect associated with the use of DFP, occurring in about 1% of the patients [48]. More common but less severe side effects are gastrointestinal symptoms, arthralgia, zinc deficiency, and fluctuating liver enzymes. Retrospective studies have shown that DFP treatment is associated with reduced cardiac morbidity and mortality [50,52,53]. DFO and DFP can be used in combination to achieve levels of iron excretion that cannot be achieved by either drug alone without increasing toxicity [54-59]. Reversal of severe iron-related heart failure with DFO and DFP combination has been reported in many patients [44,60-62]. The effect of combined therapy versus DFO monotherapy on myocardial iron overload was evaluated in a prospective, randomized, placebo controlled trial, which showed a statistically significant improvement in myocardial T2* with the combined treatment as compared with DFO and placebo treatment [63]. Combination therapy should be considered as an alternative to continuous intravenous DFO monotherapy when an intensive chelation is required.

*Deferasirox *(DFX) is a once-daily, orally administered iron chelator that a large program of clinical trials has shown to be effective in adults and children [64,65]. It received European Union marketing authorization as an orphan drug from the EMEA in 2002 and was authorized for marketing in most countries in 2006. The recommended starting dose of DFX for most patients is 20 mg/kg/day, although this can be modified to 10 or 30 mg/kg/day depending on the number of transfusions a patient is receiving and whether the therapeutic goal is to decrease or maintain body iron levels. The most frequent adverse events reported during treatment with DFX include transient, mild-to-moderate gastrointestinal disturbances and skin rash. These events rarely require drug discontinuation and most resolve spontaneously. Mild, usually nonprogressive increases in serum creatinine (generally within the upper limit of normal) has been observed in approximately a third of patients. Creatinine levels returned spontaneously to baseline in most of patients and data from up to 3.5 years of treatment in more than 1000 patients have confirmed that creatinine increase is non progressive [66]. However, cases of renal failure have been reported following the postmarketing use of DFX [67].

(S)-3'-(OH)-desazadesferrithiocin-polyether, magnesium salt is an oral once a day iron chelator expected to excrete iron mainly in the stools, evaluated in experimental models. Orphan designation of this medicine has been granted in the United States of America and Europe for treatment of chronic iron overload in patients with transfusion-dependent anemias. Recently, three main practice guidelines for the management of iron overload in thalassemia major have been published and are available online [3,68,69].

### Treatment of iron overload-related complications

#### Growth deficiency

Studies evaluating the secretion of growth hormone (GH) in patients with thalassemia major have yielded contradictory results, limiting the therapeutic use of GH to those patients proven to have GH deficiency, who may have a satisfactory response to treatment [70-72]. In cases with signs of bone toxicity from DFO a reduction of the dose, or its substitution with an oral chelator, can prevent progression of bone lesions and improve growth.

#### Delayed puberty, hypogonadism and assisted reproduction

For delayed puberty in girls, therapy may start with the administration of ethinyl estradiol (2.5-5 μg daily) for 6 months, followed by hormonal reassessment. If spontaneous puberty does not occur within 6 months, ethinyl estradiol should be used at increasing dosages (from 5-10 μg daily) for 12 months. If breakthrough uterine bleeding does not occur, a low oestrogenprogesterone hormone replacement is recommended. For delayed puberty in males, intramuscular depot-testosterone esters at a dose of 50-100 mg twice a month should be given, until complete virilisation has been achieved [72]. Topical testosterone gel can also be used [73]. When there is a lack of pubertal progression over a year or longer (arrested puberty), testosterone esters in males and oestrogenprogesterone replacement therapy in females is indicated.

In males suffering from azoospermia or asthenospermia and asking for fertility treatment, spermatogenesis may be induced by combination therapy with hCG (human chorionic gonadotrophin) and hMG (human menopausal gonadotrophin) intramuscularly or subcutaneously. Moreover, the recent advent of micromanipulation techniques such as intracytoplasmatic sperm injection (ICSI) has improved conception rates. Females with thalassemia may have primary or secondary amenorrhea, which leads to failure of the reproductive axis with chronic anovulation. Despite severe hemosiderosis, ovarian function is preserved in most patients, and they are still able to increase the estradiol level following stimulation with gonadotrophins, and furthermore produce ova. Induction of ovulation must be performed under rigorous control after a global evaluation of the patient, including detailed assessment of heart, liver function, viral infections, endocrinopathies, with particular emphasis on diabetes control and thrombophilia status [74]. Pregnant patients with thalassemia need a multidisciplinary approach involving all specialists in the medical care of thalassemia [75].

#### Hypothyroidism

Preclinical hypothyroidism is characterized by normal thyroxine (T4) and free thyroxine (FT4), normal basal TSH and TSH slightly increased after the Thyrotropin-releasing Hormone (TRH) test. A careful follow-up with an intensification of chelation therapy is required in such cases. Subclinical hypothyroidism is defined as a normal serum T4 and FT4 level with a slightly increased TSH level. It is debatable whether patients with subclinical hypothyroidism should be treated. If treatment is considered unnecessary, close monitoring is mandatory. Therapy can be recommended for patients with TSH levels greater than 10 U/ml, thyroid abnormalities, and vague symptoms attributable to hypothyroidism. In overt hypothyroidism, characterized by low T4 and FT4 values with signs and symptoms such as mental and physical sluggishness, weight gain, feeling of cold, sleepiness, bradycardia and constipation, treatment with increasing doses of L-thyroxine starting with 25 mg per day is indicated. Abnormal thyroid function may be reversible at an early stage through intensive combined chelation [76].

#### Hypoparathyroidism

Severe hypocalcemia with tetany requires intravenous administration of calcium under careful electrocardiographic monitoring, followed by oral vitamin D. In milder forms, calcitriol is the drug of choice, because of its short half-life and rapid action. A dosage of 0.25-1 μg twice daily is usually sufficient to normalize calcium and phosphate. Because of the risk of hypercalcemia and hypercalciuria, serum calcium level and 24-hour urinary calcium and phosphate measurements should be carefully monitored, especially at the beginning of treatment and if elevated doses of Vitamin D are administered.

#### Diabetes and impaired glucose tolerance

Acarbose at the dose of 100 mg (orally with breakfast, lunch and evening meals) has been used with good results for impaired glucose tolerance or non-insulin dependent diabetes mellitus and hyperinsulinism [77]. Patients with diabetes mellitus, may require daily subcutaneous injections of insulin. Since treatment of diabetes in patients with thalassemia major is an additional burden, support from doctors and psychologists is needed. Investigation of the kidney function and imaging of the fundi should be carried out to evaluate the presence and degree of diabetic complications. Intensive iron chelation therapy with DFO and DFP seems to be associated with an improvement in glucose intolerance in terms of glucose and insulin secretion, particularly in patients in early stages of glucose intolerance [78].

#### Osteoporosis

Since osteoporosis is a progressive disease, prevention is the basis of the management. No smoking, a calcium-rich diet, correction of hypogonadism by sex hormone replacement therapy and regular exercise should be recommended. Oral calcium supplements should be used with caution because of the risk of renal stones. Several bisphosphonates have been used in thalassemia patients for the treatment of osteoporosis with variable results. To date, alendronate, pamidronate, and zoledronate seem to be effective in increasing bone mineral density and normalizing bone turnover, but more controlled trials are necessary to evaluate their efficacy in reducing fracture risks in larger thalassemic populations [79].

#### Splenectomy

If the annual red cell requirement exceeds 180-200 ml/Kg of RBC (assuming that the Hct of the unit of red cells is about 75%), splenectomy should be considered, provided that other reasons for increased consumption, such as hemolytic reactions, have been excluded. Other indications for splenectomy are symptoms of splenic enlargement, leukopenia and/or thrombocytopenia and increasing iron overload despite good chelation [3].

### Bone marrow and cord blood transplantation

Bone marrow transplantation (BMT) remains the only definitive cure currently available for patients with thalassemia. The outcome of BMT is related to the pretransplantation clinical conditions, specifically the presence of hepatomegaly, extent of liver fibrosis, history of regular chelation and hence severity of iron accumulation. In patients without the above risk factors, stem cell transplantation from an HLA identical sibling has a disease-free survival rate over 90% [80]. The major limitation of allogenic BMT is the lack of an HLA-identical sibling donor for the majority of affected patients. In fact, approximately 25-30% of thalassemic patients could have a matched sibling donor. BMT from unrelated donors has been carried out on a limited number of individuals with beta-thalassemia. Provided that selection of the donor is based on stringent criteria of HLA compatibility and that individuals have limited iron overload, results are comparable to those obtained when the donor is a compatible sib [81]. However, because of the limited number of individuals enrolled, further studies are needed to confirm these preliminary findings. If BMT is successful, iron overload may be reduced by repeated phlebotomy, thus eliminating the need for iron chelation. Chronic graft-versus-host disease (GVHD) of variable severity may occur in 5-8% of individuals.

Cord blood transplantation from a related donor offers a good probability of a successful cure and is associated with a low risk of GVHD [82,83]. For couples who have already had a child with thalassemia and who undertake prenatal diagnosis in a subsequent pregnancy, prenatal identification of HLA compatibility between the affected child and an unaffected fetus allows collection of placental blood at delivery and the option of cord blood transplantation to cure the affected child [84]. On the other hand, in cases with an affected fetus and a previous normal child, the couple may decide to continue the pregnancy and pursue BMT later, using the normal child as the donor.

### Management of thalassemia intermedia

Treatment of individuals with thalassemia intermedia is symptomatic [4,85]. As hypersplenism may cause worsening anemia, retarded growth and mechanical disturbance from the large spleen, splenectomy is a relevant aspect of the management of thalassemia intermedia. Risks associated with splenectomy include an increased susceptibility to infections mainly from encapsulated bacteria (*Streptococcus Pneumoniae, Haemophilus Influenzae and Neisseria Meningitidis*) and an increase in thromboembolic events. Prevention of post-splenectomy sepsis includes immunization against the above mentioned bacteria and antibiotic prophylaxis as well as early antibiotic treatment for fever and malaise. Because of the elevated prevalence of cholelithiasis and the risks of cholecystitis in splenectomised patients, the gallbladder should be inspected during splenectomy and removed in case with or to prevent gallstones. Treatment of extramedullary erythropoietic masses, detected by magnetic resonance imaging, is based on radiotherapy, transfusions, or hydroxycarbamide. Once leg ulcer has developed, it is very difficult to manage. Regular blood transfusions, zinc supplementation and pentoxifylline, and the use of an oxygen chamber have been proposed for ulcer treatment. Hydroxycarbamide also has some benefit, either alone or with erythropoietin. Recently promising results have been obtained with platelet derived growth factor. Since patients with thalassemia intermedia have a high risk of thrombosis, exacerbated by splenectomy, it is important to be aware of thrombotic complications. Recommended treatment options include proper anticoagulation prior to surgical or other high-risk procedures, platelet anti-aggregating agents in case of thrombocytosis (platelet count higher than 700,000/mm^3^) and low molecular weight heparin in patients with documented thrombosis. Because individuals with thalassemia intermedia may develop iron overload from increased gastrointestinal absorption of iron or from occasional transfusions, chelation therapy is started when the serum ferritin concentration exceeds 300 ng/ml or when iron overload is demonstrated by direct or indirect methods [86]. Supplementary folic acid can be prescribed to patients with thalassemia intermedia to prevent deficiency from hyperactive bone marrow.

### Lifestyle and diet in beta-thalassemia

If the disease is fully compensated by ideal treatment, an individual with thalassemia major can enjoy a near-normal lifestyle and experience normal physical and emotional development from childhood to adulthood, including parenthood.

Patients with thalassemia do not have specific dietary requirements, unless they have special prescriptions. Patients already have a heavy treatment schedule and it is counterproductive to add further restrictions without the likelihood of clear benefit. During growth, a normal energy intake with normal fat and sugar content is recommended. During adolescence and adult life, a diet low in highly refined carbohydrates may be useful in preventing or delaying the onset of impaired glucose tolerance or diabetes. There is no clear evidence that a specific diet is beneficial in preventing or managing liver disease, unless at late stages. Increased iron absorption from the intestinal tract is characteristic of thalassemia. The amount depends on the degree of erythropoiesis, the Hb level and other potential independent factors. Drinking a glass of black tea with meals reduces iron absorption from food, particularly in thalassemia intermedia [85]. However, there is no evidence that iron-poor diets are useful in thalassemia major; only foods very rich in iron (such as liver, many baby foods, breakfast cereals and multivitamin preparations contain added iron, along with other vitamin supplements) should be avoided. Since many factors in thalassemia promote calcium depletion, a diet containing adequate calcium (e.g. milk, cheese, dairy products and kale) is always recommended. However, nephrolithiasis is seen in some adults with thalassemia major, and calcium supplements should not be given unless there is a clear indication; instead a low oxalate diet should be considered.

Patients with thalassemia who remain untransfused or are on low transfusion regimens have increased folate consumption and may develop a relative folate deficiency. Supplements (1 mg/day) may be given if this occurs. Patients on high transfusion regimens rarely develop this condition, and usually have no need for supplements.

Iron overload causes vitamin C to be oxidized at an increased rate, leading to vitamin C deficiency in some patients. Fifty mg of vitamin C in children <10 years and 100 mg >10 years at the time of DFO infusion may increase the 'chelatable iron' available in the body, thus increasing the efficacy of chelation. However there is currently no evidence supporting the use of vitamin C supplements in patients on DFP, DFX or combination treatment. Vitamin C may increase iron absorption from the gut, labile iron and hence iron toxicity and may therefore be particularly harmful to patients who are not receiving DFO, as iron mobilized by the vitamin C will remain unbound, causing tissue damage.

The effectiveness and safety of vitamin E supplementation in thalassemia major has not been formally assessed and it is not possible to give recommendations about its use at this time.

Patients with thalassemia should be discouraged from consuming alcohol, as it can facilitate the oxidative damage of iron and aggravates the effect of HBV and HCV on liver tissue.

In general, physical activity must always be encouraged unless there is a precise secondary medical condition. Conditions requiring special attention include splenomegaly, severe heart disease and osteoporosis.

There is no reason for patients with thalassemia to skip or delay standard recommended vaccinations. To prevent and minimize the risk of infection, immunization with the pneucococcal, *Haemophilus Influenza *and meningococcal vaccines is recommended about two weeks before splenectomy and after surgery.

It is now universally recognized that thalassemia, like other chronic diseases, has important psychological implications. The way in which the family and the patient come to terms with the disease and its treatment will have a critical effect on the patient's survival and quality of life, and a general acceptance by the patient of his/her own condition constitutes the key to normal development from childhood to adulthood. A key role for treating physicians and other health care professionals is to help patients and families to face up to the difficult demands of treatment, while maintaining a positive role.

### Therapies under investigation

Induction of HbF synthesis can reduce the severity of beta-thalassemia by improving the imbalance between alpha and non-alpha globin chains. Several compounds including 5-azacytidine, decytabine, and butyrate derivatives gave encouraging results in clinical trials [87]. These agents induce Hb F by different mechanisms not yet well defined. Their potential in the management of beta-thalassemia syndromes is under investigation.

A butyrate derivative, 2,2-Dimethylbutyric acid, sodium salt has received orphan designation for betathalassemia in the United States of America and in Europe.

The efficacy of hydroxycarbamide treatment in individuals with thalassemia is still unclear. Hydroxycarbamide has been used as experimental treatment in patients with thalassemia intermedia to reduce extramedullary masses, to increase Hb levels, and, in some cases, to improve leg ulcers. A good response, correlated with particular polymorphisms in the beta globin cluster (i.e., C>T at -158 G gamma), has been reported in individuals with transfusion dependence [88,89]. However, controlled and randomized studies are needed to establish the role of hydroxycarbamide in the management of thalassemia major.

The possibility of correction of the molecular defect in hematopoietic stem cells by transfer of a normal gene via a suitable vector or by homologous recombination is being actively investigated [90]. The most promising results in the mouse model have been obtained with lentiviral vectors [90,91]. In 2009, orphan designation was granted by the European Commission for autologous haematopoietic stem cells transduced with lentiviral vector encoding the human beta globin gene for the treatment of beta-thalassemia major and intermedia.

### Prognosis

Prognosis of thalassemia minor subjects is excellent. An increased risk for cholelithiasis, especially in association with the Gilbert mutation has been demonstrated [92]. Patients with thalassemia intermedia who do not usually have severe hemosiderosis are less prone to cardiac problems [11]. However, pulmonary hypertension, thromboembolic complications, overwhelming postsplenectomy sepsis, and the development of hepatocarcinoma may reduce survival in this group of patients. The prognosis of betathalassemia major was very grim before there was any treatment available. With no treatment, the natural history was for death by age five from infections and cachexia. The first advance in treatment was the initiation of episodic blood transfusions when the patient was having a particularly bad time. With the advent of this type of therapy, survival was prolonged into the second decade, but it soon became evident that the treatment that saved lives in children caused death from cardiac disease in adolescence or early childhood. Prognosis for individuals with betathalassemia major has dramatically improved with the advent of DFO. However, many transfusion-dependent patients continued to develop progressive accumulation of iron. This can lead to tissue damage and eventually death, particularly from cardiac disease. Advances in red cell transfusion, and the introduction of new iron chelators and chelation regimes have further prolonged survival in recent years.

Assessment of myocardial siderosis and monitoring of cardiac function combined with intensification of iron chelation have converted a once universally fatal disease to a chronic illness and an excellent long-term prognosis is expected for children who have been chelated since a very young age  [93,94].

Bone marrow transplantation is at present the only available definitive cure for patients with thalassemia major.

## Conflict of interests

The authors declare that they have no competing interests.

## Authors' contributions

RO and RG designed and wrote the paper. Both approved the final version of the manuscript, taking responsibility for the integrity of the data and the accuracy of the data analysis.
